# Defining Health in the Era of Value-Based Care: The Six Cs of Health and Healthcare

**DOI:** 10.7759/cureus.1046

**Published:** 2017-02-22

**Authors:** Mark Warren

**Affiliations:** 1 Boise Veterans Administration Medical Center, University of Washington Department of Psychiatry and Behavioral Sciences

**Keywords:** health outcomes, health expenditure, value-based care, health definition, health policy

## Abstract

Introduction: While healthcare expenditure continues to increase overall health outcomes in the United States continue to be submarginal. The changes we make in our healthcare system need to be informed by a comprehensive and actionable definition of health that can unite patients, healthcare professionals, and policymakers.

Methods: A literature review across multiple disciplines was conducted to assess a broad range of factors associated with health and well-being and based on this literature review a novel definition of health was developed.

Results: Development of a definition of health as the ability to dynamically recognize and resolve dissonance in one’s physical, mental, social and spiritual worlds via cooperation with social and spiritual connections, the medical community and with the natural world in a way that fosters and promotes harmony, resilience, and relief from suffering. This definition is then expanded into six domains (connection, communication, creativity, cooperation, cost-consciousness, and computerization) which are applicable to individuals, society and the healthcare system and which form the basis for actionable guidelines to promote measurable and sustained change on a public health scale.

Conclusion: Healthcare in the United States is changing and in order to move forward in an evidence-based and compassionate way, we need to understand what we mean by 'health' and how that definition can be operational at individual, societal, and public policy levels.

## Introduction and background

Healthcare in the US is changing and for a good reason. Despite a valiant effort, the Affordable Care Act has not ensured access to everyone, has not contained costs for individuals and families and national healthcare expenditure continues to rise sharply. Healthcare spending in the U.S. grew 5.3% in 2014, 17.5% of our GDP [1], and we continue to underperform compared to other wealthy nations [2] as we are increasingly burdened by chronic illness.

The advancement of value-based care [3] is still somewhat vague in its potential implications for our current system, especially as we grapple with improving access, assess reimbursement and measure health outcomes in a model that is still predominantly one of fee-for-service. And as our system changes, we need to realign our priorities and definitions, including our basic understanding of what we mean by health.

The World Health Organization defined health several decades ago as “a state of complete physical, mental and social well-being and not merely the absence of disease or infirmity” [4]. While this definition is appropriately broad and was lauded for its conceptualization of health as a positive entity, it has come under sharp criticism. This is partly because operationalizing the word “complete” is very difficult and the changing landscape of pathology (e.g. a shift from acute diseases to chronic diseases representing the bulk of the worldwide burden of illness) makes the definition arguably too aspirational and relegates many who are managing chronic illnesses to a camp outside of the scope of health. Finally, as Huber and colleagues have rightly pointed out, “it unintentionally contributes to the medicalization of society” [5] by encouraging a dependence on an ever expanding medical system and on medical technology with questionable benefit but substantial cost. Huber and colleagues go on the conceptualize health as “the ability to adapt and self-manage in the face of social, physical and emotional challenges” [5]. And while this is a more realistic formulation, it still lacks granularity that would foster actionable guideline formation.

I propose the following definition of health: Health is the ability to dynamically recognize and resolve or meaningfully manage dissonance in one’s physical, mental, social and spiritual worlds via cooperation with social and spiritual connections, the medical community and with the natural world in a way that fosters and promotes harmony, resilience, and relief from suffering.

As an expansion of the above definition, I outline six components (connection, communication, creativity, cooperation, cost-consciousness, and computerization) which are essential to achieving health by this definition and which provide a framework for understanding the individual, social and public health applications of such a definition.

## Review

### The six Cs of health and healthcare

### Connection

We live in a world of fragmented attachment and we have understood for years that this is unhealthy and potentially dangerous. From studies of anaclitic depression (first described by Rene Spitz in work with infants separated from their mothers) and failure to thrive syndromes to more current shifting paradigms of addiction as a disorder where an unhealthy connection is at least partially implicated, we recognize that human connection is vital to health. In spite of a proliferation of diagnostic codes within the International Classification of Diseases (ICD-10), there is actually very little that captures the idea of social disconnectedness or loneliness as a driver of illness. However, there is ample literature showing how loneliness is pro-inflammatory during acute stress [6], is correlated with widespread reduced regional white matter density in areas important for empathy and self-efficacy [7] and can predict mortality similarly to other known medical risk factors such as cardiovascular disease [8-9]. Further, Thomas Joiner argues compellingly that when an individual has acquired the ability to lethally self-injure and this is coupled with a sense of thwarted belongingness (i.e. social disconnectedness) and perceived burdensomeness, the risk for suicide is significantly elevated [10].

But not just any connection will do. Compassionate connections that influence and help shape core beliefs of competence and “a subjective sense of belonging and intimacy” [11] are the most important for health. “Social connection is linked to health, well-being, social competence, and increased survival as well as a prosocial orientation toward the world, helping to create a highly beneficial and mutually reinforcing set of variables” [11].

The primary guideline application to this aspect of health is a continued focus on access to care for everyone that is compassionate and encourages competent engagement and shared ownership of an individual patient’s health.

### Communication

The ability to communicate effectively is integral to health. For instance, we know that childhood victims of abuse are at risk for delayed language skill development [12] and also that hearing impairment in children can be associated with increased stress, reduced quality of life and lower self-esteem compared to children who hear normally. [13]

Healthcare professionals (at least doctors) are notorious for using medical jargon and often raising more questions than we answer. Effective communication between a physician and patient has been shown to actually improve health outcomes [14]. This seems straightforward but often underemphasized in the clinical encounter. Yolanda Partida reminds us that “physicians need to understand that the health world is a foreign country to many Americans and pay closer attention to understanding the language patients use and how they draw meaning from what they hear” [15].

From a guideline perspective, we need to prioritize direct practitioner-patient communication more in our training with individual patients, but also between various healthcare settings and between pharmacies to help ensure safer medication regimens.

### Creativity

The ability to explore, maintain a sense of wonder, to have fun and enjoyment in life (the axiomatic “pursuit of happiness”) is indispensable to health in this model. The ability to be creative in the marketplace and be involved in a community that values different opinions and tastes is a marker of a healthy society.

In the healthcare arena, creativity has the potential to lead to significant improvements. By examining what other countries do well (and what they don’t do well); by reducing our dependence on inordinately expensive pharmaceutical research and development and making a more concerted effort to promote preventative and lifestyle interventions, we could help reduce costs and improve outcomes. For instance, why don’t we look at the places on earth where people are living the longest and healthiest lives such as Okinawa, Sardinia and Loma Linda, California (CA)? [16].

It is now old news that diet and other lifestyle interventions can have a dramatic and lasting effect on disease processes previously thought to be irreversible. Caldwell Esselstyn [17], Dean Ornish and others [18] have demonstrated that prevention models and actually putting money into researching and funding lifestyle interventions could have a transformative influence on U.S. healthcare economics and outcomes.

Ornish and colleagues remind us that the worldwide leading causes of mortality are now chronic non-communicable diseases (NCDs); diseases such as cardiovascular disease, cancer, respiratory diseases, and diabetes. They highlight the “now overwhelming evidence that lifestyle factors […] are key proximal factors in the pathogenesis and incidence of NCDs” and that “it is imperative that we finally and systematically address the underlying causes of LRDs [lifestyle-related diseases] rather than superficially treating symptoms” [18]. This includes broad policy interventions prioritizing raising awareness of the importance of lifestyle medicine, prioritizing prevention and lifestyle research and interventions, re-prioritizing aspects of medical curriculum and training and fostering more collaboration with researchers, clinicians, and policymakers [18].

As a practical (and potentially inflammatory) guideline example, we need to take a hard look at why the government continues to subsidize the production of foods that have been shown to be highly correlated (if not causative) of many disease processes we are plagued with in the Western world. As the Physician’s Committee for Responsible Medicine (PCRM) has pointed out, “the U.S. Department of Agriculture (USDA) supports agricultural producers through a variety of programs that tend to favor, either directly or indirectly, the production of unhealthful foods. These are the same foods that are implicated in the diseases that have steadily increased over the decades and now impose a significant burden on Americans” [19]. We need to take a hard look at our government subsidy programs and skewed funding of expensive interventions over sustainable and more simple and widely applicable common-sense interventions (e.g. diet, exercise, stress reduction, etc.).

### Cooperation

Cooperation presupposes at least a rudimentary connection and a certain level of communication. On an individual level, obstinate adherence to a ‘my way or the highway’ relational approach is clearly disastrous.

The development of initiatives such as the collaborative care model for patients with depression and diabetes and/or cardiovascular disease, Cardiovascular Outcomes for People using Anticoagulation Strategies (COMPASS) has demonstrated that cooperation within the healthcare system and shared responsibility for care can have demonstrated positive health outcomes [20]. We are seeing integrated care models (co-location of mental health practitioners in primary care settings for example) improve access and this will likely lead to improved outcomes and cost savings. Looking at incorporating more community health center work in our system is also worthy of exploration. And as we move to a more convenient and user-friendly model of care, we can begin to engage more sincerely in the all-important task of shared-decision making with our patients, prioritizing their personhood over and above their patient-hood [21] further reinforcing the positive connection.

### Cost-consciousness

When personal debt and living in the reality of our nation’s indebtedness seems to be the norm, it can be easy to lose track of just how problematic our healthcare spending is. But overspending without being able to afford it is unhealthy for families and unhealthy for nations. With soaring costs, the inability to keep a trusted physician and millions still under or uninsured, we have clearly not fixed the problem with recent legislation. And despite our out of control spending, we live with the unsettling reality that we are not getting enough return on investment. Ranking 37th in the world is simply unacceptable [22].

Part of cost containment involves a careful analysis of where the money actually goes and closing the gap between delivery of care and business aspect of that care delivery. Customers (patients) and providers are often blind to the actual price of healthcare and there is wide variability in what the actual cost could be for a particular service. This is a financial transaction without parallel in the broader marketplace and the fact that healthcare costs are so mystical and difficult to decipher should be unacceptable. There is nothing wrong with fair compensation for practitioners (especially considering the substantial training costs, both direct costs and those lost to productive work time. Add to this the high liability, stress and technical expertise needed to do a good job and it makes sense that it should be appropriately compensated). And I’m not arguing against a reasonable profit for those involved in the business of healthcare (including the pharmaceutical and insurance industries). But is there a way to make the cost to the consumer more transparent and can we look at ways of streamlining our model so that we can contain cost more effectively? Can we make the business side of medicine more transparent, more accountable, especially to the patients we serve?

Part of the issue in healthcare that affects cost as well is the ever-increasing burden on practitioners to conform to policymaker demands that seem to be divorced from the reality of the day to day functions of the clinic and hospital. And, as Bray and Althausen point out, “[…] the passage of recent healthcare legislation now places the orthopedic surgeon in a precarious position by reducing reimbursement while increasing ‘penalty-driven’ cost-of-care control measures. The government’s mandate demanding lower-cost care regardless of the legal care standard continues to place even more burden on orthopedic practices without any indication that ‘tort reform’ will ever become part of the equation” [23]. This is not limited to orthopedic care. Simply demanding better care and imposing fines in a top-down fashion without a more substantive discussion about where we are actually going and what tools we need to get there is not the solution. Increasingly, physicians need to be dialoguing with policymakers and industry about the day to day realities of delivering medical care and also have some protection under the law.

Cleanliness of process and making access and billing more straightforward should be a priority. Partly underlying the reason why many in our nation use our emergency departments so regularly could be simplicity. You just show up. No appointment, no prior authorizations, no hoops to jump through with regard to insurance coverage. And many people are willing to accept the backside consequences of bills, collectors, etc. as long as they can just be seen in a (relatively) straightforward fashion.

### Computerization

Technology certainly is beneficial on the number of levels. But overuse of technology can have its drawbacks and there is a risk that overuse can actually be detrimental to health. An example is a research showing that increasing sedentary behavior (largely as a function of our engagement and dependence on technology or what I would call “over-computerization”) is associated with increases in all-cause mortality [24].

While technology can bolster communication, I would argue that we are probably the most disconnected (or perhaps more appropriately stated-superficially connected) generation yet. We have endless ways to communicate but the evidence that this is leading to lasting and healthy connection is lacking. And our disconnection from the natural world and increasing global urbanization have been associated with increased levels of depression. Recent research has shown that experiencing nature via an extended walk can reduce rumination and activation in an area of the brain thought to be associated with “self-focused behavioral withdrawal” [25] and may help link the phenomenon of increased urbanization and increasing mental illness burden.

From a healthcare standpoint, we need to meet patients where they are at and use technology appropriately in their care, but also beware of implicitly and explicitly endorsing overuse which may have unintended consequences.

## Conclusions

If we are going to stem the tide of healthcare spending and improve our health as individuals and as a nation, we need to begin with a vision and definition of health that is appropriately broad yet granular enough to contain actionable, understandable and empirically driven components. The six Cs of healthcare (connection, communication, creativity, cooperation, cost-consciousness and computerization) contain the seeds to both understand what constitutes true health from an empirically-verifiable standpoint and casts a vision for uniting policymakers with patients and clinicians to tackle one of the most fundamental challenges we currently face as a nation: ensuring that our wealth, expertise, and ingenuity are leveraged to guarantee the opportunity for each of our citizens to enjoy optimal health. And if we are going to do this, we need to understand health in a way that decentralizes the problem and diffuses the responsibility of our nation’s health to all of us, not just the medical community. As such, I reiterate what I propose as an actionable and realistic definition of health: Health is the ability to dynamically recognize and resolve or meaningfully manage dissonance in one’s physical, mental, social and spiritual worlds via cooperation with social and spiritual connections, the medical community and with the natural world in a way that fosters and promotes harmony, resilience, and relief from suffering.

Dennis Kraus ended a recent paper with this admonition. “I advise all of my colleagues to be kind and compassionate to your patients; help them to understand their disease; assist them in making treatment choices, and return them to the best quality of life you are able. This will be the ultimate manifestation of living in a patient-centric universe” [26]. At a time when our physician workforce and physician workforce-in-training are under significant strain [27-28] and as we think about the monumental task of simultaneously reigning in healthcare costs and improving the health of our nation, we need to clarify where we are going. We need to resist the urge to reflexively escalate regulations and demand change and improvement with the threat of punitive action and confront the flawed core belief that policymakers alone can solve the problem. We need to avoid the urge to think that one piece of sweeping legislation (or undoing sweeping pieces of legislation with other sweeping pieces of legislation) in and of itself will fix the problem. When we connect patients, healthcare professionals and policymakers with a common vision of what can reasonably be expected from our system we will open the way for creative and compassionate development of both policy and intervention at a reasonable cost with better outcomes.
